# **Baby and us: Community-based, Feasibility Trial of a Psychosocial Intervention for New Parents and their Infants**

**DOI:** 10.1007/s10935-022-00685-0

**Published:** 2022-07-28

**Authors:** Joshua Harwood, Leire Fernández, Valentina Vallejo, Crispin Day

**Affiliations:** 1 Department of Psychology, Institute of Psychiatry, Psychology and Neuroscience, King’s College London, London, UK; 2 Centre for Parent and Child Support, South London & Maudsley NHS Foundation Trust, London, UK; 3 The School of Psychology, Universidad de Talca, Talca, Chile; 4 The School of Psychology of the Pontificia, Universidad Católica de Chile, Santiago, Chile; 5 Harwood Child Psychology, London, UK

**Keywords:** Parenting, Parenting programme, Infancy, Early intervention, Parent child relationships, Peer support

## Abstract

Infancy is a critical period during which major developmental transformations occur. Early parenting is one of the strongest influences on infants’ immediate and longer-term outcomes. The transition to parenting can be demanding and stressful for mothers and fathers. This paper reports results from a feasibility study of the Empowering Parents Empowering Communities Baby and Us programme, an 8-week, universal, peer-led parenting programme for new parents living in socially disadvantaged communities. This study is a quasi-experimental, one arm, no control group study, assessing the feasibility and acceptability of Baby and Us. Programme participants (n = 158) completed standardised self-report measures of parent goal attainment, self-efficacy, knowledge about parenting, mental wellbeing, parental confidence, and programme acceptability. We found that recruiting parents from disadvantaged backgrounds was feasible (96% of programmes recruited sufficient parents to proceed, mean = 6.6 parents per programme); parent goals closely matched the aims of the programme; programme completion was high (74%), and self-report measurement completion rates were in line with other large scale community delivered parenting programmes; parents rated the programme as highly satisfactory; and they reported significant improvements in their mental wellbeing, confidence, parenting skills, self-efficacy, and goal attainment. These results provide important data to conduct a full-scale trial of Baby and Us.

## Introduction

Parenting has a profound influence on development (Allen, 2011; Yoshikawa, 2010; Zeanah, 2009). Emotional, behavioural and regulatory problems in infants and very young children are common and associated with problematic motor, language, cognitive and relational development (DeGangi et al., 2000; Skovgaard et al., 2008). Early infant parenting requires rapid skill acquisition, heightened emotional and relational sensitivity, and practical adjustment (Bornstein, 2002). Parenting infants typically improves parents’ emotional fulfilment, life satisfaction and social roles, but are also associated with frequent negative emotional states (Feeney et al., 2001).

Most early parenting interventions aim to improve infant development, positive parenting, parent-infant relationships, and prevent early childhood problems (Barlow et al., 2010; NICE, 2013). The strongest evidence for early parenting interventions stems from intensive nurse-led home visiting programmes, such as Nurse Family Partnership and Maternal Early Child Sustained Home-visiting Evans et al., 2015; Kemp et al., 2011; Molloy et al., 2020; Olds et al., 2007). Typically, these are provided to individual mothers experiencing specific high-risk indicators, such as first-time and young mothers, or those experiencing multiple risk factors and low psychological, social and economic resources. These intensive programmes usually begin before birth and continue to age two, frequently involving a schedule of weekly to monthly visits provided by highly qualified nurses (Beatson et al., 2021). The programmes are conveniently delivered in the home, offering tailored individual care over a substantial duration. Systematic reviews (Kendrick, Elkan & Hewitt, 2000; Molloy et al., 2020) suggest that outcomes are inconsistent and vary across programmes, with some interventions being more effective for specific ‘higher risk’ subgroups, such as young mothers (Olds et al., 2007). Given the intensive format and extended duration of these interventions, less is known about programme cost-effectiveness, though UK evidence indicates that immediate effects may not have cost-effectiveness advantages relative to existing universal services (Molloy et al., 2020).

Systematic reviews and meta-analyses consistently demonstrate the impact on child development, family outcomes and cost-effectiveness of group-format parenting programmes delivered outside of the home during middle childhood (Michelson et al., 2013; NICE, 2013). This programme format is usually delivered over 8–12 weekly sessions involving 8–12 parents within a group. Notwithstanding the broader outcome evidence, concern has been expressed about the ability of such programmes to engage higher-risk parents and be delivered at sufficient scale to meet family need, particularly for disadvantaged families and families from minority ethnic groups.

Evidence for the use of group parenting programmes using similar methods and formats during infancy is less strong (Barlow et al, 2010). For example, Barlow et al (2016) conclude that universal and selective parenting programmes may be effective in improving the emotional and behavioural adjustment of infants and toddlers but further evidence is required about the specific benefits and longer term effects.

With mixed success, task-sharing, peer-led and paraprofessional approaches have been developed as methods to increase the availability and acceptability of parenting interventions, particularly for low income and socially excluded families (Day et al., 2012; Olds et al., 2002; Peacock et al., 2013).

Empowering Parents Empowering Communities (EPEC) *Being a Parent* is a low cost, peer-led, community-based, group-format parenting programme originally developed for parents of children, aged 2–11 years. Its group-based format is intended to build social support between participants, optimise impact on parent and child outcomes, and lower unit cost. EPEC is delivered in community locations within targeted disadvantaged communities. Within the targeted community, an open access approach is typically used, rather than formal referral or selection of high-risk individuals. The peer-led format is associated with high levels of parent engagement, acceptability and reduced stigma (Day et al., 2012; Day, Kearney & Squires, 2017). Randomised control trial and field evidence shows that EPEC *Being a Parent* successfully reaches socially disadvantaged parents of children aged 2–11 years, is highly acceptable, and produces significant improvements in child behaviour, positive parenting and parental concerns, when delivered by peer parent group leaders (PGLs) recruited from within target populations (Day et al., 2012; Day et al., 2020) ,

Given the potential of group-format parenting programmes and the use of peer-led delivery, the current evaluation sought to examine the initial feasibility of a version of the EPEC programme, called *Baby and Us* (B&U; Penney et al., 2016), specifically designed to engage and improve outcomes for parents and infants in the first year of life. The aim of the B&U programme was to improve parental self-esteem and the parent-infant relationship, increase parenting confidence, and encourage positive parenting. In this paper, we report the results of a two-phased pilot evaluation examining the feasibility and acceptability of B&U, whilst investigating effect size change to determine the parameters for a future large scale definitive trial.

The study addressed the feasibility of the newly developed Baby and Us (B&U) programme. The study aimed to understand rates of recruitment, intervention completion and self-report measurement completion rates. It aimed to uncover how successfully B&U reaches disadvantaged parents and parents from minority ethnic groups and measure how consistent B&U programme aims are with parents’ stated goals. The study aimed to preliminarily measure how satisfied parent participants were with the B&U programme and also to assess preliminary effect size change on parent wellbeing, confidence, self-efficacy, and goal attainment, with the purpose of informing the study design of a future full-scale randomised controlled trial.

## Methods

### Design

This study used a quasi-experimental, one arm, no control group design organised in two phases. The intervention and overall study design were identical across both phases, the only difference between phase one and phase two is the battery of measures used. The study’s measures were modified prior to the second phase to examine a wider range of outcome domains. Parental self-report measures were collected at the first meeting session of any B&U intervention group (Time 1) and in the final session of a B&U intervention group – 8 weeks later (Time 2). This was identical in both phases of the programme.

### Participants

We successfully recruited 159 parents to B&U programmes. One programme only recruited one parent and was cancelled. The final sample size for analysis was n = 158 parents. Families were eligible for inclusion if a primary parental caregiver (“parent”) had self-identified difficulties in managing their infant, aged between 0–12 months, including parental stress, parent-infant relationship difficulties, interaction difficulties, and social isolation. The inclusion criteria was outlined using parent friendly language on all recruitment information including flyers and information given to children’s centres to discuss with parents. Parents self-identifying as struggling were given further information about the programme and a telephone number they could call for further information. We planned to exclude families from the study if (1) there was an identified serious developmental disorder in the child, (2) the parent was unable to read and write in English, (3) the parent had a serious post-natal mental health disorder, (4) the facilitators had significant safeguarding concerns, and (5) the parent was not currently living at home with the index child. To increase the accessibility of the programme, we established no specific inclusion or exclusion criteria for the type or severity of problems, other than the fact that the parents were seeking help with managing their baby. Parents were screened for the eligibility criteria during an initial pre-programme introductory session.

### Procedure

Parents were recruited through word of mouth, posters, referrals and face to face contacts. Parents registered to attend by emailing or calling on the provided details, or by letting a member of staff know at their local children’s centre. Parents attended a pre-programme introductory session, lasting for two hours and at the same time and location as the subsequent full B&U programme. The aim of the introductory session was to give parents greater understanding of the aims of B&U, ask questions and agree participation. A cohort of 11 trained and accredited parent facilitators, working in pairs, delivered the 25 B&U programmes. Programmes were held in 17 community sites in the London Boroughs of Southwark (*n* = 5), Lambeth (*n* = 1), Croydon (*n* = 6) and Newham (*n* = 1), as well as in Southend (*n* = 11). Around 85% of venues were located in areas of significant multiple deprivation, including income, housing quality and crime (IOD, 2019).

### Intervention

The Baby & Us “programme” is a manualised, targeted, early parenting programme consisting of eight weekly, two-hour “sessions” for between 6–8 parents, of infants 0–12 months old (Penney et al., 2016). Developed by Dr Day and colleagues, it uses a variety of interactive learning methods to improve parent knowledge about infant development, communication, care routines, interaction and stimulation skills, parental coping, confidence, reflective functioning, sensitivity, bonding, and to develop social support networks. Programme completion is defined as attending five or more sessions (Day et al., 2012). Facilitators are local parents who receive 60 hours of accredited initial training, followed by fortnightly supervision and observed practice from specialist parenting professionals (Day et al., 2017). All programmes were delivered in children’s centres in the respective locations. See Table 1 for B&U programme details and session content.

**Table 1:** B U Programme Sessions and Topics

Session	Session topics
Session 1: Processing the birth of your baby	Getting to know each other
	Goals for parent and baby
	Describing your birth
	Recovery from birth
Session 2: Being good enough	‘Good enough’ vs. ‘perfect’ parent
	Taking care of yourselves
	Coping with crying
Session 3: You and your baby’s feelings	Acknowledging babies’ feelings
	How parent feelings are connected with our babies’ feelings
	Managing family and baby stress
Session 4: Family and care routines	Creating and managing daily and family routines
	Feeding styles and routines
	Sleeping styles and routines
Session 5: Getting to know your baby	My personality and my baby’s personality
	Understanding different moods and states
	Types of crying and communication
Session 6: Connecting with your baby	Babies’ interactions with parents
	Descriptive commenting
	Parentese
	Types of touch
Session 7: Keeping safe	Keeping babies safe
	Managing stress and parent difficulties
Session 8: Review and support	Reviewing the course & knowing where to get support
	Ending and celebration

### Outcome measures

Feasibility measures:

We measured ***programme attendance*** using detailed intervention attendance records. In these, we noted the date, name of parent, and whether they were present or not. To determine ***reach***, we collected detailed demographic data, including parent age, gender, ethnicity, language, education, work status, disability status, lone parent status, child age, gender and disability status.

To measure ***Programme acceptability and satisfaction*** we used the Treatment Acceptability Rating Scale (TARS; Day et al., 2012): The TARS is a 9-item self-report questionnaire measured on a 4-point Likert scale with 3 additional open ended questions at the end. The TARS was specifically developed for assessing (i) EPEC parenting knowledge, skills and confidence (TARS KSC − 4 items yield total score 4–16 ), (ii) programme satisfaction and quality (TARS SQ − 5 items yield total score 5–20), and (iii) 3 free-text items about participant experience. Higher TARS scores on the first 9 items indicate greater knowledge gain/programme satisfaction. Example TARS KSC items include “Did the programme increase your understanding of positive parenting” and “ Has the programme made you feel more confident in being an effective parent?”. Examples TARS SQ items includes “Overall how satisfied are you with the programme?” and “Did the group leaders relate to the group effectively”.

In both phases, we measured parent mental well-being and functioning using the **Warwick Edinburg Mental Well-Being Scale** (WEMWBS; Tennant, et al., 2007), a 14-item scale, each with 5 response categories. The WEMWBS has good internal consistency, a = 0.91 (Tennant, et al., 2007). We also used the **My Parenting Goals** (MPG; Harwood et al., 2018), a two-item measure of parental goal achievement where parents write two, free text goals. Goal achievement was rated on 0-100 scale, with 100 representing complete achievement.

In Phase 1 only, we measured parent self-efficacy using a **Tool to Measure Parenting Self-Efficacy** (TOPSE; Bloomfield & Kendall, 2007). Two TOPSE subscales, Self-Acceptance and Learning/Knowledge were used, each consisting of 6 items per subscale, rated using an 11-point Likert scale. A higher score indicated greater efficacy. Self-acceptance, a = 0.89, learning/knowledge, a = 0.81 (Bloomfield & Kendall, 2007). Example items include “I know I am a good enough parent”, “As a parent I can take most things in my stride” and “I am able to learn and use new ways of dealing with my child”.

In Phase 2 only, we measured perceived parental self-efficacy using the **Karitane Parenting Confidence Scale** (KPCS; Crncec, Barnett & Matthey, 2008). The KPCS is a 15-item scale, scored from 0 to 3, with higher scores indicating greater self-efficacy. The KCPS showed adequate internal consistency (a = 0.89, Crncec, Barnett & Matthey, 2008), example items included “I am confident about feeding my baby”, “I understand what my baby is trying to tell me” and “I can make decisions about the care of my baby”.

### Data analysis

Measures were scored and missing data treated as per the relevant measure manuals. Pairwise deletion was practised for all feasibility analysis. Frequency counts and percentages were calculated for categorical variables. No significant differences were found between the demographic characteristics of Time 2 questionnaire completers and non-completers. The data were tested for normality and homoscedasticity and data was suitable for parametric testing. Paired sample t-tests were conducted.

Acceptability was examined using TARS descriptive statistics and programme completion rates. A coding scheme was developed to categorise the parent’s primary goal (see Table 2). The first author (JH) created the coding template by reading the parents goals and grouping them based on distinct categories. Overlapping categories were merged. Broad categories with too many entries were broken down into more specific categories and those with too few entries (*n* < 3) were merged into larger categories where appropriate or labelled as idiosyncratic.

## Results

1. Recruitment and intervention/measurement completion.

We successfully recruited 159 parents with a baby aged 0 to 12 months to 25 Baby and Us (B&U) programmes in 17 different venues between January 2015 and July 2019. We delivered 25 programmes, and one programme had only a single parent in attendance so was not completed. This final programme delivery was not included in any analyses and therefore the sample size for analysis is *n* = 158 parents. The mean number of parents per completed programme was 6.6 (range = 2 to 13 parents).

Overall programme completion rate was high (74.0%). The mean number of programme sessions attended was 5.5 (*SD* = 2.2) out of a possible 8. There were no significant differences between the demographic characteristics of programme completers and non-completers.

Overall, 158 (100%) parents provided Time 1 data. *n* = 94 parents (59.5%) provided any Time 2 data. Time 2 completion rates were somewhat lower for measures later in the questionnaire pack (range 48.3% − 59.5%).

2) How successfully does Baby and Us reach disadvantaged parents and parents from minority ethnic groups?

Table 2 summarises the demographic characteristics of parents attending the B&U parenting programme. The vast majority were mothers (*n* = 155, 98.1%) and of White British ethnicity (*n* = 61, 52.6%). The other common parent ethnicities were Non-British White (*n* = 17, 14.7%), Black African (*n* = 9, 7.8%) and Black African-Caribbean (*n* = 6, 5.2%). Infant mean age was 20.2 weeks, 50.0% aged between 13–27 weeks, and 25.0% younger than 13 weeks. The education profile of parents attending B&U groups was mixed. Attending parents had below national average levels of university education (44.9% B&U vs. 55% for females nationally - ONS, 2019) and slightly higher levels of leaving school with no formal qualifications than average (20.4% vs. 18% nationally – The Children’s Commissioner, 2019). Approximately 24% of parents across the study had English as a second language. Only 25% were owner occupiers of their house compared to 63% nationally (ONS, 2011) and 50% in London (ONS, 2011)

**Table 2:** Demographic characteristics of parents and infants attending B&U programmes

Demographic Characteristics (%)%			
**Parent Gender (n = 158)**		**Parent Ethnicity (n = 116)**	
Male	1.9	White British	52.6
Female	98.1	Black and minority ethnic group	47.4
**Target Infant Gender (n = 157)**		**Highest Educational Level (n = 118)**	
Male	45.2	University education completed	44.9
Female	54.8	Left school aged 16 with qualifications	6.8
		**Participating in Paid Employment (n = 117)**	64.1
Demographic variable	Mean (SD)	Demographic variable	
**Age of Parent**	31.3 (5.8) years	**English as a second language (n = 108)**	24.1
**Age of infant**	20.2 (9.5) weeks		

Where sample size allowed for greater insight, it was clear that B&U programmes succeeded at attracting parents from minority ethnic groups at rates higher than is representative of their communities. See Table 3. Note, two out of the five local authority areas in this study (40%) only ran one programme therefore there is insufficient data to make comparisons.

**Table 3:** Proportion of parents from Black and Minority Ethnic groups attending B&U programmes compared to the local authority hosting the programme

Local Authority	Black and Minority Ethnic representation in area (%) (ONS, 2011)	Black and Minority ethnic representation in Baby and Us programmes (%).
Southend on Sea	8.4	25.6
Southwark	45.8	65.0
Croydon	44.9	57.8
England Total	21.2	47.4

3) How consistent are Baby and Us programme aims with stated parent goals?

Table 4 displays the primary goals that parents reported at the start of the intervention and compares them to B&U stated programme aims. The most common being to gain knowledge about general parenting (*n* = 30, 21.9%), improving knowledge about routines e.g. feeding/sleeping (*n* = 23, 16.8%) and increasing parent’s social network (*n* = 20, 14.6%). Fifteen parents (10.9%) stated their primary goal as improving their own mental health, which was not directly addressed as a programme aim. The closest matched programme aim was to help parents process the impact of the birth experience, however zero parents noted this as their primary goal of attending. Three out of 11 (27.3%) B&U programme aims were not corroborated by parent reported goals: (1) To help mothers and fathers to gain an understanding of their own, their baby’s and their partner’s feelings and how these can be interdependent, (2) To help strengthen relationship with partner, (3) To help parents process the impact of the birth experience

**Table 4:** Primary My Parenting Goal categories of parents attending B&U

Goal category	N (%)	Relevant B&U programme aim
Improve knowledge about parenting	30 (21.9)	To understand how to stimulate their baby using singing, touch and physical play. Increase maternal and paternal sensitivity to their baby’s cues.
Improve knowledge about infant routines/feeding/sleeping	23 (16.8)	To help mothers and fathers understand their baby’s crying/sleeping/feeding and to feel more confident managing the practicalities of their baby’s routine.
Improve understanding of infant and parent-infant communication/relationship	18 (13.1)	To help mothers and fathers communicate with their baby. To build attachment between mothers and babies, and fathers and babies. To acquire an understanding of their baby’s temperament
Improve personal confidence	19 (13.9)	Give mothers and fathers more confidence in their role.
Increase social network	20 (14.6)	To help parents develop friendships with other parents which are mutually supportive.
Improve parental mental health	15 (10.9)	To help parents process the impact of the birth experience.
Other*	12 (8.8)	
	total *n* = 137	

*Parent goal was written in a way that did not contain enough information to categorise or *n* < 3 so did not form category. Examples included “save money for holiday”, “graduate from university”, “how to prioritise the things”, “me”

4) How satisfied were parent participants with the B&U programme?

Parents reported high levels of programme satisfaction with the quality of the programme and the competence, skills and qualities (SQ) of the Baby and Us group leaders (TARS SQ Time 2 mean = 19.2, range = 16–20). See Table 5 for individual item responses.

**Table 5:** Post B&U programme results on the Treatment Acceptability Rating Scale for programme satisfaction and quality (TARS-SQ)

TARS programme Satisfaction and Quality (SQ) (*n* = 79)	Not at all - *n* (%)	A little - *n* (%)	Quite a lot - *n* (%)	A great deal - *n* (%)
Competent B&U parent group leaders	0 (0.0)	0 (0.0)	22 (27.8)	57 (72.2)
Satisfaction with B&U programme and group leaders	0 (0.0)	0 (0.0)	17 (21.5)	62 (78.5)
Appropriate content covered by B&U programme	0 (0.0)	0 (0.0)	7 (8.9)	72 (91.1)
B&U group leaders related effectively to parent participants	0 (0.0)	0 (0.0)	12 (15.5)	67 (84.8)
B&U group leaders motivated participant parents	0 (0.0)	1 (1.3)	8 (10.3)	69 (88.5)

5) What were the reported changes in parent wellbeing, confidence, self-efficacy and goal attainment?

**Table 6:** Parent and infant outcomes following the B&U intervention

Measure	Time 1 Mean (SD)	Time 2 Mean (SD)	Statistic	P value	Effect Size (d)
Phase 1 and 2					
MPG Goal 1 (*n* = 75)	38.2 (27.5)	79.4 (19.2)	t=-13.1	0.000	1.6
MPG Goal 2 (*n* = 72)	37.9 (28.5)	77.2 (22.4)	t=-10.2	0.000	1.2
WEMWBS (*n* = 88)	48.1 (8.6)	54.2 (6.9)	t=-7.5	0.000	0.8
Phase 1 only					
TOPSE self-acceptance (*n* = 56)	40.6 (6.9)	46.4 (4.8)	t=-7.2	0.000	1.0
TOPSE Learning and knowledge (*n* = 60)	48.0 (8.3)	53.7 (5.3)	t=-6.3	0.000	0.8
Phase 2 only					
KPCS (*n* = 30)	29.1 (11.0)	32.8 (11.4)	t=-5.0	0.000	0.7

Parents reported improvements in their goals, in mental wellbeing, self-acceptance, learning, knowledge and parenting confidence, all with a large effect sizes (See Table 6). The vast majority of parents rated that they increased their understanding and skills in positive parenting, increased parental confidence and felt more equipped to use their learning in practice (See Table 7). TARS KSC Time 2 mean = 14.1, range = 4–16.

**Table 7:** Post B&U programme results on the Treatment Acceptability Rating Scale for parent knowledge, skills and confidence (TARS-KSC)

TARS Knowledge, Skills and Confidence (KSC)	Not at all - *n* (%)	A little - *n* (%)	Quite a lot - *n* (%)	A great deal - *n* (%)
Better understanding of positive parenting (*n* = 78)	1 (1.3)	3 (3.8)	31 (39.7)	43 (55.1)
Better skills in positive parenting (*n* = 79)	1 (1.3)	3 (3.8)	31 (39.2)	44 (55.7)
Increased parental confidence (*n* = 79)	1 (1.3)	5 (6.3)	31 (39.2)	42 (53.2)
Expectations of using learned B&U content in practice (*n* = 79)	2 (2.5)	3 (3.8)	25 (31.6)	49 (62)

## Discussion

This study evaluated the feasibility, acceptability and outcomes of the EPEC Baby and Us (B&U) programme, a peer-led parenting intervention designed to improve outcomes for parents of babies, living in socially disadvantage communities.

The findings suggest that it is feasible to recruit parents to (B&U) parenting groups. Only one group (4%) did not recruit sufficient parents to warrant continuing. The mean number of parents per B&U group (6.6) is in line with the target group size of between 6–8 parents. It is fewer than the mean number of parents attending a variant of EPEC for parents with children aged 2–11 years (mean = 9.3, Day et al, 2012b), however smaller group sizes are necessary in B&U groups due to the infants being present in sessions. Reduced group size has pros and cons. Larger groups have greater reach, are potentially more cost effective and offer more social networking opportunities. Smaller group sizes offer parents more individual attention from facilitators. Smaller B&U groups may increase cost per participant, but overall these costs may be offset by infants being present in the sessions and onsite childcare not being provided. Full cost analysis will be an important part of a future definitive trial.

The B&U programme completion rate (74%) was in line with mean completion rates (72%) found in a systematic review of behavioural parent training programmes (Chacko et al., 2016). Completion rates are an indication of programme acceptability, suggesting B&U is acceptable to the majority of parents. Acquiring data about non-completion in future studies may provide further insight and understanding into reasons for disengagement and potential remedial strategies.

B&U participant self-report measurement completion rates were similar or higher than many other community delivered parenting programmes (Lindsay & Strand, 2013, Lindsay & Totsika, 2017, Lindsay et al., 2011). However, measurement completion rates were lower than those achieved in a randomised control trial of EPEC Being a Parent, designed for parents of older children (Day et al., 2012b). The current study was delivered in routine settings with fewer research staff to promote and assist measure completion and no financial renumeration was available to parents. Time 2 measures were completed at the end of the final programme session. Programme non-completion and non-attendance at the final programme session was therefore a key feature in non-completion of measures. A full trial would need appropriate research staff and resources to increase data yield to rates comparable to previous EPEC trials (Day et al., 2012 b). Missing data is problematic for validity as, for example, dissatisfied parents may have been more likely to leave the intervention prematurely. Due caution in interpretation is therefore warranted and a further full-scale trial utilising intention to treat analysis will be beneficial in the future.

EPEC’s peer-led programme model is designed to increase reach and engagement of parents from socially disadvantaged and minority communities. Baby and Us programmes were largely located in highly disadvantaged communities – over 70% were in the bottom third most deprived areas, characterised by low employment, high crime, low education, poor housing quality and low incomes. Location was subject to venue availability and it was not always possible to ensure venues are delivered in the most disadvantaged locations. Where sample size allowed analysis to take place, B&U groups succeeded at attracting parents from minority ethnic groups at rates higher than community base rates. This is important considering the multiple barriers to engagement that parents from ethnic minorities can encounter (Forehand & Kotchick, 1996) although caution in interpretation is warranted due to low sample size per location. Rates of English as a second language were high, highlighting the attractiveness of B&U for families who might otherwise be marginalised.

The education profile of parents attending B&U groups was mixed. Whilst the groups did attract disadvantaged parents with high rates of low education, they were also attractive to university educated parents, albeit at reduced rates compared to national education levels. Parent level of owner occupier status was 40–50% lower than national rates. Some of this variation can be explained by the lower average rates of owner occupier status of Black and Minority Ethnic groups across the UK and also the relatively lower rates of owner occupier status of people aged 20–35 (ONS, 2011), the main demographic attending B&U groups.

Parents came to the B&U programme to achieve a range of goals, the most common being to gain knowledge about general parenting (21.9%), routines e.g. feeding, sleeping (16.8%), and increasing parent’s social network (14.6%). Comparisons suggested a good match between parent goals and intervention aims. The only significant discrepancy was related to parents’ goal to improve mental health and well-being. Interestingly, analysis suggested that programme participation may result in improvements in mental wellbeing. The programme has been subsequently amended to further strengthen parental mental health content. Pilots are underway to assess the benefits of B&U for mothers experiencing perinatal mental health difficulties.

Parents who provided Time 2 TARS data reported high levels of user satisfaction. Rates of dissatisfaction were minimal and consistent with other studies, accredited EPEC parent group leaders were perceived as very effective and motivational.

Programme impact was not a primary aim of this feasibility study and the design cannot reliably assess for impact, however these preliminary results suggest potential improvements in parenting goals, well-being, self-efficacy, parental confidence, knowledge and skills that require further evaluation within a definitive trial. The magnitude of effects detected suggest that sample size calculation for a definitive trial should assume medium to large effect sizes. The authors were cautious about participant measurement burden, particularly pertinent considering the high levels of English as a second language. A full trial would benefit from measures of cost effectiveness, as well as potentially parent infant bonding, parental reflective functioning and parenting stress. Considering the recorded measurement completion rates, measurement burden will need to be carefully monitored in a definitive trial.

At this stage, these effects are promising and, though achieved within an uncontrolled feasibility study, add to the limited and inconsistent evidence about early parenting group-based interventions (Evans et al. 2015; Jones et al., 2016; Pontopidanet al., 2016; Tsivos et al., 2015). The results also add to the growing evidence derived from EPEC on the acceptability of parent-led parenting interventions (Day et al., 2012a, b; Day et al., 2020).

The evaluation of routine B&U programmes in routine community settings provides robust external validity. This feasibility trial did not assess randomisation. Due to non-randomisation, positive outcomes cannot be unequivocally attributed to programme participation. Results suggest B&U has a positive impact on early parenting characteristics that promote and protect early infant development that warrant further examination in a randomised trial incorporating measures of infant development and longer-term follow-up.

The study had limitations. The study design was such that causal impact of the programme could not be measured. Similarly, a subsequent design will need to account for the impact of running the different programmes with different facilitators. Whilst the majority of parents reported high levels of satisfaction with the programme and facilitators, we did not have sufficient statistical power to determine the individual impact of different facilitators. Due to limited resources, we were not able to follow up with parents who dropped out. It is therefore not clear at this stage the reasons for parents dropping out, and therefore it is possible the results are biased towards participants who really valued the intervention and therefore completed. At this stage we can hypothesise why some parents dropped out, including not being able to commit to 8 weeks, work and family commitments and not finding the programme helpful. In addition to this, some parents did not complete outcome measures despite completing the programme. Reasons for this include facilitators running out of time, parents needing to leave before the end of the session for personal reasons and not being able to attend session 8, where follow up self-report questionnaires were administered. Future studies will require greater research assistant input to increase measurement completion rates and follow up rates, and systematically document reasons for non-completion.

These findings provide preliminary support for the B&U programme as an acceptable, peer-led intervention that has the potential to promote parent’s mental well-being, confidence and self-efficacy during infancy. Results contribute to the limited evidence base for early intervention group-parenting programmes for parents and infants. Importantly the study shows that parents from socially disadvantaged communities successfully engage in the B&U programmes, reporting high levels of programme satisfaction and acceptability.

## References

[CR1] Allen, G. (2011). Early Intervention: The Next Steps. Retrieved from: https://www.gov.uk/government/uploads/system/uploads/attachment_data/file/284086/early-intervention-next-steps2.pdf

[CR2] Barlow, J., Smailagic, N., Ferriter, M., Benett, C., & Jones, H. (2010). Group-based parent-training programmes for improving emotional and behavioural adjustment in children from birth to three years old. *The Cochrane database of systematic reviews 17*(3), Article CD003680-CD003680. doi: 10.1002/14651858.CD003680.pub2 10.1002/14651858.CD003680.pub2 PMC4164454 20238324

[CR3] Barlow, J., Bergman, H., Kornør, H., Wei, Y., & Bennett, C. (2016). Group-based parent training programmes for improving emotional and behavioural adjustment in young children. *Cochrane Database of Systematic Reviews*, (8) 10.1002/14651858.CD003680.pub3 PMC6797064 27478983

[CR4] Beatson R, Molloy C, Perini N, Harrop C, Goldfeld S (2021). Systematic review: An exploration of core componentry characterizing effective sustained nurse home visiting programs. Journal of Advanced Nursing.

[CR5] Bloomfield L, Kendall S (2007). Testing a parenting programme evaluation tool as a pre- and post-programme measure of parenting self-efficacy. Journal of Advanced Nursing.

[CR6] Bornstein, M. H. (Ed.). (2005). *Handbook of parenting: volume 4 social conditions and applied parenting*. Psychology Press

[CR7] Chacko A, Jensen SA, Lowry LS, Cornwell M, Chimklis A, Chan E, Pulgarin B (2016). Engagement in behavioral parent training: Review of the literature and implications for practice. Clinical Child and Family Psychology Review.

[CR8] Črnčec R, Barnett B, Matthey S (2008). Karitane Parenting Confidence Scale: Manual. Sydney South West Area Health Service.

[CR9] Day, C., Kearney, C., & Squires, F. (2017). Art, Science and Experience of Peer Support: Learning from the Empowering Parents, Empowering Communities Programme.International Journal of Birth and Parent Education, Vol *4*(2)

[CR10] Day C, Michelson D, Thomson S, Penney C, Draper L (2012). The Empowering Parents, Empowering Communities: a peer-led parenting programme. Child and Adolescent Mental Health.

[CR11] Day, C., Michelson, M., Thomson, S., Penney, C., & Draper, L. (2012b). Evaluation of a peer-led parenting intervention for child behaviour problems: A community-based randomised controlled trial. *British Medical Journal*, *344*, doi: 10.1136/bmj.e1107 10.1136/bmj.e1107 22416059

[CR12] Day C, Nicoll J, Harwood J, Kendall N, Kearney L, Kirkwood J (2020). Transforming Children’s Lives: EPEC Scaling Programme. NESTA/Department of Digital Culture Media & Sport.

[CR13] DeGangi GA, Breinbauer C, Roosevelt JD, Porges S, Greenspan S (2000). Prediction of childhood problems at three years in children experiencing disorders of regulation during infancy. Infant Mental Health Journal: Official Publication of The World Association for Infant Mental Health.

[CR14] Evans S, Davies S, Williams M, Hutchings J (2015). Short-term benefits from the Incredible Years Parents and Babies Programme in Powys. Community Practitioner.

[CR15] Feeney JA, Hohaus L, Noller P, Alexander RP (2001). Becoming Parents: Exploring the Bonds between Mothers, Fathers and their infants.

[CR16] Forehand R, Kotchick BA (1996). Cultural diversity: A wake-up call for parent training. Behaviour Therapy.

[CR17] Hackney M, Braithwaite S, Radcliff G (1996). Postnatal depression: the development of a self-report scale. Health Visit.

[CR18] Harwood, J., Kendall, N., & Day, C. (2018). *My parenting goals: An idiographic measure of parent progress*. EPEC/SLAM

[CR19] IOD (2019). Indices of Deprivation. Retrieved from http://imd-by-postcode.opendatacommunities.org/imd/2019

[CR20] Jones CH, Erjavec M, Viktor S, Hutchings J (2016). Outcomes of a Comparison Study into a Group-Based Infant Parenting Programme. Journal of Child and Family Studies.

[CR21] Kemp L, Harris E, McMahon C, Matthey S, Vimpani G, Anderson T, Zapart S (2011). Child and family outcomes of a long-term nurse home visitation programme: a randomised controlled trial. Archives of disease in childhood.

[CR22] Kendrick D, Elkan R, Hewitt M, Dewey M, Blair M, Robinson J, Brummell K (2000). Does home visiting improve parenting and the quality of the home environment? A systematic review and meta-analysis. Archives of disease in childhood.

[CR23] Lindsay G, Strand S (2013). Evaluation of the national roll-out of parenting programmes across England: The parenting early intervention programme (PEIP). Bmc Public Health.

[CR24] Lindsay G, Strand S, Davis H (2011). A comparison of the effectiveness of three parenting programmes in improving parenting skills, parent mental-well-being and children’s behaviour when implemented on a large scale in community settings in 18 English local authorities: The parenting early intervention pathfinder (PEIP). Bmc Public Health.

[CR25] Lindsay G, Totsika V (2017). The effectiveness of universal parenting programmes: The CANparent trial. BMC Psychology.

[CR26] Molloy C, Beatson R, Harrop C, Perini N, Goldfeld S (2021). Systematic review: effects of sustained nurse home visiting programs for disadvantaged mothers and children. Journal of Advanced Nursing.

[CR27] NICE (National Institute of Health and Care Excellence) (2013). *Social and emotional well-being for children and young people.* London: NICE. Retrieved from: https://www.nice.org.uk/advice/lgb12/chapter/what-nice-says

[CR28] Olds DL, Robinson J, O’Brien R, Luckey DW, Pettitt LM, Henderson CR, Talmi A (2002). Home visiting by paraprofessionals and by nurses: a randomized, controlled trial. Paediatrics.

[CR29] Olds DL, Sadler L, Kitzman H (2007). Programs for parents of infants and toddlers: recent evidence from randomized trials. Journal of child psychology and psychiatry.

[CR30] ONS (Office for National Statistics) (2011). Retrieved from https://www.nomisweb.co.uk/reports/localarea?compare=E06000033

[CR31] ONS (Office for National Statistics) (2019). Retrieved from https://www.gov.uk/government/collections/statistics-on-higher-education-initial-participation-rates

[CR32] Peacock S, Konrad S, Watson E, Nickel D, Muhajarine N (2013). Effectiveness of home visiting programs on child outcomes: a systematic review. BMC public health.

[CR33] Penney C, Wilson C, Kearney L, Adewole C, Day C (2016). Baby and Us Empowering Parents, Empowering Communities Parenting Group Manual.

[CR34] Pontoppidan M, Klest SK, Sandoy TM (2016). The Incredible Years Parents and Babies Program: A Pilot Randomized Controlled Trial. Plos One.

[CR35] Skovgaard AM, Olsen EM, Christiansen E, Houmann T, Landorph SL, Jørgensen T, CCC 2000* Study Group (2008). Predictors (0–10 months) of psychopathology at age 1½ years–a general population study in The Copenhagen Child Cohort CCC 2000. Journal of Child Psychology and Psychiatry.

[CR36] Tennant R, Hiller L, Fishwick R, Platt S, Joseph S, Weich S, Stewart-Brown S (2007). The Warwick-Edinburgh Mental Well-being Scale (WEMWBS): development and UK validation. Health and Quality of Life Outcomes.

[CR37] The Children’s Commissioner (2019). Retrieved from https://www.childrenscommissioner.gov.uk/wp-content/uploads/2019/09/cco-briefing-children-leaving-school-with-nothing.pdf

[CR38] Tsivos Z, Calam R, Sanders MR, Wittkowski A (2015). A pilot randomized controlled trial to evaluate the feasibility and acceptability of the Baby Triple P Positive Parenting Programme in mothers with postnatal depression. Clinical Child Psychology and Psychiatry.

[CR39] Yoshikawa, H. (2010). The foundations of lifelong health are built in early childhood

[CR40] Zeanah, C. H. Jr. (2009). The scope of infant mental health. CH Zeanah (Ed.), Handbook of Infant Mental Health

